# Early-Onset Diabetes in Lahore and Kasur Districts of Punjab: A Hospital-Based Study

**DOI:** 10.3390/jcm15218390

**Published:** 2026-10-28

**Authors:** Muhammad Khurram, Muhammad Sohail Afzal, Muhammad Tayyab, Qaisar Raza, Muhammad Arif Nadeem Saqib, Syed Qasim Raza

**Affiliations:** 1 Institute of Biochemistry and Biotechnology, Faculty of Bio-Sciences, University of Veterinary and Animal Sciences, Lahore 54000, Pakistan; 2 Department of Life Sciences, School of Science, University of Management & Technology, Lahore 54770, Pakistan; sohail.afzal@umt.edu.pk; 3 Department of Food Science and Human Nutrition, Faculty of Bio-Sciences, University of Veterinary and Animal Sciences, Lahore 54000, Pakistan; qaisar.raza@uvas.edu.pk; 4 Department of Health Sciences Technology, National Skills University, Islamabad 44000, Pakistan

**Keywords:** early-onset diabetes, type 1 diabetes, type 2 diabetes, parental consanguinity, family history, Pakistan

## Abstract

**Background:** Early-onset diabetes is increasing in South Asia, but the distribution and correlates of diabetes types among young Pakistani patients are poorly characterized. **Methods:** This cross-sectional study at five tertiary-care hospitals in Lahore and Kasur, Pakistan, included 856 patients with type 1 or type 2 diabetes diagnosed at or before 40 years of age, grouped by age at diagnosis (1–10, 11–20, 21–30, and 31–40 years). Factors associated with clinically assigned type 1 diabetes were examined using Firth penalized logistic regression adjusted for age at diagnosis, gender, BMI category, first-degree family history, parental consanguinity, and physical activity. **Results:** Type 1 diabetes was assigned to 255 participants (29.8%), including 97.6% and 73.6% of those diagnosed at 1–10 and 11–20 years, compared with 21.9% and 10.4% of those diagnosed at 21–30 and 31–40 years. BMI and blood pressure were higher with later diagnosis, whereas glycemic control was uniformly poor (mean HbA1c, 9.1%; *p* = 0.356). Compared with diagnosis at 1–10 years, the adjusted odds of type 1 diabetes were lower with diagnosis at 11–20 years (aOR, 0.0767; 95% CI, 0.0083–0.3134), 21–30 years (aOR, 0.0083; 95% CI, 0.0009–0.0335), and 31–40 years (aOR, 0.0034; 95% CI, 0.0004–0.0144) and in females (aOR, 0.35; 95% CI, 0.24–0.51), and higher with parental consanguinity (aOR, 1.98; 95% CI, 1.36–2.91) and underweight BMI (aOR, 1.95; 95% CI, 1.04–3.70). First-degree family history (aOR, 0.94; 95% CI, 0.61–1.48) and physical activity (aOR, 1.35; 95% CI, 0.92–1.98) were not associated with diabetes type. The optimism-corrected area under the curve was 0.846. **Conclusions:** Clinically assigned diabetes type differed sharply by age at diagnosis and was independently associated with gender, parental consanguinity, and underweight BMI.

## 1. Introduction

Diabetes mellitus remains one of the leading non-communicable diseases worldwide, and its prevalence continues to rise [1,2]. The burden is especially high in South Asia, where diabetes develops at a younger age than in Western populations; urbanization, nutrition transition, physical inactivity, and intergenerational metabolic vulnerability have all been linked to this earlier onset [3,4]. National surveys in Pakistan have documented a high prevalence of diabetes among adults [5,6,7], and a substantial proportion of new cases now arises in childhood, adolescence, and early adulthood [8,9]. Diagnosis before 40 years of age prolongs lifetime exposure to hyperglycemia and leads to earlier accumulation of microvascular and macrovascular complications during the most economically productive decades of life [9,10,11].

Early-onset diabetes is heterogeneous and includes autoimmune, insulin-resistant, and monogenic forms. Distinguishing type 1 from type 2 diabetes in young people is often difficult on clinical grounds, because phenotypes overlap in populations with increasing adiposity and changing environmental exposures [10,12,13]. Early-onset type 2 diabetes is characterized by more rapid decline in β-cell function, a more aggressive clinical course, and a greater lifetime burden of complications than type 2 diabetes diagnosed later in life [8,11,14], and the recognition of distinct endotypes within type 1 diabetes has highlighted the need for accurate etiological classification [15]. South Asians develop diabetes at a younger age and at a lower BMI than White populations, with greater insulin resistance attributed to more visceral and ectopic fat and lower skeletal muscle mass [3,16]. Together with dietary change, low cardiorespiratory fitness, and socioeconomic factors, these features make BMI thresholds derived from European populations unsuitable for risk stratification [17,18].

Family history is one of the most easily obtained risk factors for early-onset diabetes. In early-onset type 2 diabetes, diabetes in a parent or sibling is associated with younger age at diagnosis, greater insulin secretory defects, and earlier treatment failure [8,9], whereas in type 1 diabetes familial aggregation reflects both HLA and non-HLA genetic susceptibility [19]. In South Asian multigenerational households, heritable metabolic risk is compounded by shared dietary patterns, physical activity, and psychosocial stressors [3,4]. Parental consanguinity, which is common in Pakistan, is a less explored factor: marriages between biological relatives increase the likelihood of homozygosity for rare recessive variants, including those that cause monogenic diabetes [20,21], and genetic evaluation of consanguineous families with atypical phenotypes has proved valuable in Pakistani children [21].

Lifestyle exposures are modifiable even in people with a genetic predisposition. Sedentary behavior, unhealthy diet, smoking, and sleep disruption are associated with adverse glucose metabolism and cardiometabolic deterioration [4,14,22,23]. Active smoking and exposure to second-hand smoke are associated with an increased risk of type 2 diabetes [24,25], and sleep duration has a U-shaped association with the risk of type 2 diabetes [22,23]. These factors have not been examined together in early-onset diabetes in Pakistan. Studies that combine clinical, familial, anthropometric, and behavioral data are therefore likely to describe early-onset diabetes more accurately than studies that rely on diagnostic labels alone.

Pakistan, with one of the highest diabetes burdens in the world, marked urban–rural differences, and a high prevalence of consanguineous marriage, is an important but under-researched setting for studies of early-onset diabetes [6,7,20]. This study therefore aimed to describe the demographic, familial, anthropometric, behavioral, and clinical characteristics of patients with diabetes diagnosed at or before 40 years of age in Lahore and Kasur, Punjab, across age-at-diagnosis groups from childhood to young adulthood, and to identify factors associated with clinically assigned type 1 diabetes, in order to inform context-specific screening and prevention strategies.

## 2. Materials and Methods

### 2.1. Study Design, Setting, and Participants

This multicenter cross-sectional study was conducted at five public tertiary-care hospitals in Punjab, Pakistan: Jinnah Hospital, Shaikh Zayed Hospital, Sir Ganga Ram Hospital, Mayo Hospital in Lahore, and Baba Bulleh Shah Hospital in Kasur. The study was designed to characterize clinical heterogeneity across age-at-diagnosis groups among patients with early-onset diabetes, rather than to estimate population prevalence, and is reported in accordance with the Strengthening the Reporting of Observational Studies in Epidemiology (STROBE) guideline [26].

Patients were recruited consecutively from the diabetes outpatient clinics of the participating hospitals. Patients with type 1 or type 2 diabetes first diagnosed at or before 40 years of age were eligible if sufficient interview and medical record information was available to determine diabetes type and the study variables; participants could not be older than 40 years at enrollment. Patients with neonatal diabetes, with diabetes and deafness suggestive of maternally inherited diabetes and deafness, or with diabetes diagnosed after 40 years of age were excluded. Age at diagnosis was ascertained from the interview and medical records, recorded in completed years, and grouped as 1–10, 11–20, 21–30, and 31–40 years to allow comparison of childhood-, adolescent-, and young-adult-onset disease within a single regional sample. The minimum sample size was calculated using the formula n = Z^2^ × p(1 − p)/d^2^ × DEFF, assuming a proportion of 50% (which maximizes the required sample size), an absolute precision of 5%, a 95% confidence level (Z = 1.96), and a design effect of 2 to allow for clustering of participants within the five hospitals, which gave a minimum of 769 participants. We additionally confirmed that the achieved sample was adequate for the multivariable logistic regression using the criteria of Riley et al.: with 10 regression parameters, an outcome proportion of 0.298, and a conservative anticipated Cox–Snell R^2^ of 0.106 (15% of its maximum), at least 801 participants (239 events) were required [27]. Of 950 patients assessed for eligibility, 94 were excluded (40 with incomplete records, 30 who declined participation, and 24 who met exclusion criteria), leaving 856 participants.

### 2.2. Diagnosis and Classification of Diabetes

Diabetes had been diagnosed according to American Diabetes Association criteria: fasting plasma glucose ≥ 126 mg/dL, HbA1c ≥ 6.5%, or random plasma glucose ≥ 200 mg/dL with symptoms of hyperglycemia [28]. The primary outcome, diabetes type (type 1 or type 2), was the clinical diagnosis recorded by the treating physician on the basis of insulin requirement from diagnosis, diabetic ketoacidosis at presentation, and body habitus. Islet autoantibodies, C-peptide, and genetic testing were not routinely available for classification; diabetes type therefore represents a clinically assigned label rather than an etiologically confirmed subtype.

### 2.3. Data Collection and Family Characteristics

Data were collected by trained staff through structured interviews in Urdu, using a questionnaire based on the WHO STEPwise instrument that was pretested in 20 patients not included in the analysis, together with review of medical records and direct anthropometric, clinical, and laboratory measurements. Sociodemographic characteristics comprised age at enrollment, age at diagnosis, gender, district (Lahore or Kasur), ethnicity, highest education (no formal schooling, primary school, secondary school, high school, college/university, or postgraduate degree), marital status (married, unmarried, or divorced), and occupation (government employee, non-government employee, self-employed, other, unemployed, or student). Family history was recorded as the list of blood relatives with diabetes (mother, father, siblings, grandparents, and uncles or aunts), from which we derived diabetes in any relative and diabetes in a first-degree relative (parent or sibling), together with the type of diabetes in affected relatives (type 1 or type 2). Parental relationship was recorded as first cousins, second cousins, third cousins, other blood relation, or no family relation.

For regression analyses, highest education was grouped into low (no formal schooling or primary school), medium (secondary or high school), and high (college/university or postgraduate degree) levels, with one participant who declined to answer treated as missing; marital status was grouped as married versus unmarried (including one divorced participant); and occupation was grouped as unemployed versus all other occupational categories, with students (n = 77) and participants who did not answer (n = 56) excluded from this comparison (n = 723). Parental relationship was dichotomized as consanguineous (any blood relation between the parents) versus non-consanguineous.

### 2.4. Tobacco Exposure, Dietary Intake and Physical Activity of Recruited Subjects

Lifestyle characteristics were self-reported at enrollment using structured questions referring to a typical week during the preceding 3 months; they therefore describe current behavior rather than behavior at the time of diagnosis. Tobacco exposure was recorded as current tobacco smoking, current smokeless tobacco use, and ever tobacco use. Dietary intake was recorded as sugary beverages (servings/week), fruit and vegetable consumption (days/week), and junk food consumption (never, rarely, once a month, once a week, twice a week, or daily). Physical activity was recorded as the number of days per week with walking and with sports; participants who reported walking or sports on at least one day per week were classified as physically active for regression analyses. The daily duration of activity was not recorded consistently and was not analyzed. Sleep duration was classified as <6, 6–8, or >8 h per day, and usual bedtime as early (8:00–10:00 pm) or late (after 10:30 pm). Experience of stress was recorded as yes or no.

### 2.5. Clinical and Anthropometric Characteristics

Height and weight were measured by trained staff with participants in light clothing and without shoes, and BMI was calculated as weight in kilograms divided by height in meters squared. In participants aged 18 years or older at enrollment, BMI was categorized using WHO cut-offs for Asian populations as underweight (<18.5 kg/m^2^), normal (18.5–22.9 kg/m^2^), overweight (23.0–24.9 kg/m^2^), or obese (≥25.0 kg/m^2^) [17]. In the 50 participants younger than 18 years, BMI-for-age z-scores were calculated from the WHO 2007 growth reference [29] and categorized as thinness (<−2 SD, analyzed with underweight), normal (−2 to +1 SD), overweight (>+1 to +2 SD), or obesity (>+2 SD); age in months was calculated from the date of birth when available and otherwise from age in completed years. Systolic and diastolic blood pressure were measured in the seated position with a standard sphygmomanometer after 5 min of rest, as the mean of two readings taken 1–2 min apart. Hypertension was defined as systolic blood pressure ≥140 mmHg, diastolic blood pressure ≥ 90 mmHg, or use of antihypertensive medication. Antidiabetic treatment (insulin only, tablets only, or both), antihypertensive drugs, and cardiovascular preventive drugs (aspirin or statins) were recorded for all participants.

### 2.6. Laboratory Measurements

Venous blood was collected after an overnight fast of at least 8 h for fasting blood glucose. Fasting and random blood glucose were measured by the glucose oxidase–peroxidase method on a Selectra ProS analyzer (ELITech Group, Puteaux, France), and HbA1c was measured in EDTA whole blood by high-performance liquid chromatography (D-10, Bio-Rad Laboratories, California, United States). Specimens were transported at 4 and analyzed within 24 h of collection. Because these measurements were obtained at enrollment, they reflect current glycemic control rather than values at diagnosis.

### 2.7. Statistical Analysis

Continuous variables are presented as mean ± SD and categorical variables as n (%). Differences across the four age-at-diagnosis groups were assessed using Welch’s one-way analysis of variance, because group sizes were unequal (42 to 356 participants) and Levene’s test indicated unequal variances for several variables; the Kruskal–Wallis test was also applied as a check against non-normality and gave the same conclusions. When the overall test was significant, pairwise comparisons were made using the Games–Howell procedure (Supplementary Table S1). Categorical variables were compared using the chi-square test, or Fisher’s exact test when more than 20% of expected cell counts were below 5 (with Monte Carlo simulation, 100,000 replicates, for tables with more than two rows and two columns). Participants with clinically assigned type 1 and type 2 diabetes were compared using Welch’s *t*-test and the chi-square test.

Logistic regression was used to identify factors associated with clinically assigned type 1 diabetes, with type 2 diabetes as the comparison group. Because only one participant in the 1–10-year group had type 2 diabetes, conventional maximum-likelihood estimates for age at diagnosis were affected by near-separation; all models were therefore fitted using Firth’s penalized-likelihood logistic regression, with profile penalized-likelihood 95% CIs and *p*-values [30]. Unadjusted odds ratios (ORs) were first estimated for age at diagnosis, gender, BMI category, first-degree family history, parental consanguinity, education, physical activity, occupation, marital status, stress, tobacco use, and hypertension. Variables with *p* < 0.20 in univariable analysis were considered candidates for the multivariable model [31]; because purely data-driven selection has known limitations [32], final covariates were then chosen on a priori grounds. Marital status was excluded because it is largely a proxy for age; stress and hypertension were excluded because they are likely to be consequences of diabetes or its treatment; and tobacco use was excluded because it was uncommon (6.0%) and almost absent among participants diagnosed before 20 years of age, so that its distribution was largely determined by age. Education and occupation did not meet the screening threshold. In addition, occupation was recorded at enrollment and largely reflected age and gender roles, so it may be a consequence of diabetes rather than a confounder. The multivariable model therefore included age at diagnosis, gender, BMI category, first-degree family history, parental consanguinity, and physical activity, and yielded adjusted ORs (aORs) with 95% CIs. Reference categories were 1–10 years, male gender, normal BMI, no first-degree family history, no consanguinity, and physical inactivity.

The multivariable model included 856 participants, 255 of whom had type 1 diabetes, and 10 estimated parameters (three for age at diagnosis, three for BMI category, and one each for gender, family history, consanguinity, and physical activity), giving 25.5 events per parameter, above the commonly recommended minimum of 10 [33]. Multicollinearity was assessed using generalized variance inflation factors. Calibration was assessed with the Hosmer–Lemeshow test (10 groups), interpreting a non-significant result as no evidence of poor calibration rather than as proof of good fit, and with a calibration plot and the calibration slope and intercept [34]. Discrimination was assessed by the area under the receiver operating characteristic curve (AUC) with DeLong 95% CIs, and sensitivity and specificity were reported at the threshold that maximized the Youden index. Optimism in the AUC and calibration slope was estimated by bootstrap resampling with 1000 replicates [35], and the AUC of a model containing age at diagnosis alone was compared with that of the full model using DeLong’s test.

Seven sensitivity analyses were performed: (i) with the 1–10- and 11–20-year groups combined into a single reference category (≤20 years); (ii) excluding the 50 participants younger than 18 years at enrollment; (iii) excluding participants whose clinical label was discordant with their treatment (type 1 diabetes treated with oral agents only, n = 66); (iv) restricted to characteristics that are fixed or precede diagnosis (age at diagnosis, gender, first-degree family history, and consanguinity); (v) replacing first-degree family history with family history in any relative; (vi) adding education to the final model; and (vii) adding both education and occupation, with students and participants who did not report their occupation retained as separate categories, supplemented by a complete-case analysis of participants with known employment status. All tests were two-sided, and *p* < 0.05 was considered statistically significant. Analyses were performed in R version 4.6.1 (R Foundation for Statistical Computing, Vienna, Austria) using the packages logistf, pROC, ResourceSelection, car, and anthroplus.

### 2.8. Ethical Considerations

The study was conducted in accordance with the Declaration of Helsinki and was approved by the Institutional Review Committee for Biomedical Research, University of Veterinary and Animal Sciences, Lahore, Pakistan (letter No. 368/IRC/BMR; 14 May 2025), with administrative approval from each participating hospital. Written informed consent was obtained from all participants before enrollment; for participants younger than 18 years, written consent was obtained from a parent or legal guardian, with verbal assent from the participant. Personal identifiers were removed from the dataset before analysis.

## 3. Results

### 3.1. Sociodemographic and Family Characteristics

A total of 856 participants were included, with age at diagnosis of 1–10 years in 42 (4.9%), 11–20 years in 140 (16.4%), 21–30 years in 356 (41.6%), and 31–40 years in 318 (37.1%) (Table 1). The mean age at diagnosis was 26.6 ± 8.2 years, and the mean age at enrollment was 32.5 ± 7.4 years; mean age at enrollment increased from 20.5 ± 6.7 years among participants diagnosed at 1–10 years to 37.9 ± 3.2 years among those diagnosed at 31–40 years. Most participants resided in Lahore (719 [84.0%]) and were Punjabi (844 [98.6%]), and 510 (59.6%) were female; neither district (*p* = 0.672) nor gender (*p* = 0.158) differed across age-at-diagnosis groups.

Overall, 255 participants (29.8%) had clinically assigned type 1 diabetes and 601 (70.2%) had type 2 diabetes, and diabetes type differed markedly across age-at-diagnosis groups (*p* < 0.001). Type 1 diabetes accounted for 97.6% of cases diagnosed at 1–10 years and 73.6% of those diagnosed at 11–20 years, whereas type 2 diabetes accounted for 78.1% and 89.6% of cases diagnosed at 21–30 and 31–40 years, respectively. Marital status and occupation also differed across groups (both *p* < 0.001), largely in parallel with age: the proportion of unmarried participants declined from 69.0% in the 1–10-year group to 49.3%, 7.9%, and 2.2% in successive groups, and the proportion of students from 35.7% to 28.6%, 4.8%, and 1.6%. Highest education also differed across groups (*p* = 0.010). A family history of diabetes in any relative was reported by 719 participants (84.0%) and in a first-degree relative by 613 (71.6%); first-degree family history increased with older age at diagnosis (31.0%, 49.3%, 77.5%, and 80.2%; *p* < 0.001). Among participants with an affected relative, the relative had type 1 diabetes in 146 (20.3%) and type 2 diabetes in 564 (78.4%).

Parents were consanguineous in 432 participants (50.5%): first cousins in 302 (35.3%), second cousins in 35 (4.1%), third cousins in 22 (2.6%), and other blood relations in 73 (8.5%), while 424 (49.5%) reported no family relation. Parental relationship differed across age-at-diagnosis groups (*p* = 0.019), with the proportion of consanguineous parents declining from 59.5% in the 1–10-year group to 55.0%, 51.7%, and 45.9% in successive groups, mainly because of fewer first-cousin unions (42.9% vs. 27.0% in the youngest and oldest groups).

### 3.2. Lifestyle Characteristics

Tobacco smoking was reported by 51 participants (6.0%) and increased from 0% in the 1–10-year group to 2.1%, 5.3%, and 9.1% in successive groups (*p* = 0.007), whereas smokeless tobacco use (41 [4.8%]; *p* = 0.542) and ever tobacco use (71 [8.3%]; *p* = 0.051) did not differ significantly (Table 2). Vegetable consumption differed across groups (*p* < 0.001), with daily consumption declining from 50.0% in the 1–10-year group to 37.1%, 23.0%, and 15.1% in successive groups, as did fruit consumption (*p* = 0.007), with daily consumption of 26.2% and 26.4% in the two youngest groups compared with 17.1% and 11.3% in the two oldest. Junk food consumption also differed across groups (*p* = 0.016), with 128 participants (15.0%) consuming junk food at least once a week, whereas sugary beverage intake did not differ (*p* = 0.402).

Walking frequency differed across groups (*p* = 0.003), with the proportion reporting no walking rising from 52.4% in the 1–10-year group to 55.0%, 65.4%, and 69.2% in successive groups. Sports activity was rare, with 823 participants (96.1%) reporting none (*p* = 0.551), and 309 participants (36.1%) were classified as physically active. Sleep duration differed across groups (*p* = 0.004): sleep of less than 6 h per day was absent in the 1–10-year group and was reported by 5.0%, 5.1%, and 9.7% of participants in successive groups, whereas bedtime did not differ (*p* = 0.183).

### 3.3. Clinical Characteristics

Experience of stress was reported by 666 participants (77.8%), with no difference across groups (*p* = 0.341) (Table 3). Antidiabetic treatment differed markedly across groups (*p* < 0.001): overall, 322 participants (37.6%) used insulin only, 342 (40.0%) tablets only, and 192 (22.4%) both; insulin only was used by 95.2% of participants in the 1–10-year group but by 17.6% of those in the 31–40-year group, whereas tablets only were used by 2.4% and 59.4%, respectively. Antihypertensive drugs were used by 81 participants (9.5%), more often in the 21–30- and 31–40-year groups (10.7% and 11.9%) than in the 1–10- and 11–20-year groups (4.8% and 2.1%) (*p* = 0.005), and hypertension was present in 224 participants (26.2%), increasing from 14.3% and 11.4% in the two youngest groups to 28.4% and 31.8% in the two oldest (*p* < 0.001). Cardiovascular preventive drugs were used rarely, and their use did not differ across groups (aspirin, 13 [1.5%]; statins, 8 [0.9%]; *p* = 0.507).

Anthropometric and blood pressure measures increased with older age at diagnosis (all *p* < 0.001). Mean BMI rose from 19.5 ± 3.0 kg/m^2^ in the 1–10-year group to 21.4 ± 4.8, 24.5 ± 4.7, and 25.9 ± 4.8 kg/m^2^ in successive groups, and all pairwise differences in BMI were significant (Supplementary Table S1). The proportion of participants classified as obese increased from 4.8% to 17.9%, 37.4%, and 55.7%, whereas underweight was most frequent in the 11–20-year group (22.9%). Weight rose from 46.9 ± 10.2 kg to 55.8 ± 14.5, 64.8 ± 12.3, and 67.9 ± 12.3 kg, and height from 154.6 ± 13.4 to 162.3 ± 9.5 cm between the youngest and oldest groups. Systolic blood pressure increased from 111.5 ± 15.4 to 125.9 ± 14.0 mmHg and diastolic blood pressure from 71.4 ± 9.7 to 80.4 ± 8.3 mmHg between the youngest and oldest groups. In contrast, glycemic control was uniformly poor and did not differ across groups, with an overall fasting blood glucose of 167.2 ± 48.3 mg/dL (*p* = 0.520), random blood glucose of 287.7 ± 106.4 mg/dL (*p* = 0.460), and HbA1c of 9.1 ± 2.3% (*p* = 0.356).

### 3.4. Factors Associated with Clinically Assigned Type 1 Diabetes

In univariable analyses (Table 4), the odds of type 1 diabetes decreased markedly with older age at diagnosis relative to 1–10 years (OR, 0.0998; 95% CI, 0.0110–0.3949 for 11–20 years; OR, 0.0102; 95% CI, 0.0011–0.0389 for 21–30 years; and OR, 0.0042; 95% CI, 0.0005–0.0167 for 31–40 years; all *p* < 0.001). These very small ORs reflect the near absence of type 2 diabetes in the reference group (1 of 42 participants). Type 1 diabetes was also associated with underweight BMI (OR, 2.11; 95% CI, 1.28–3.49), parental consanguinity (OR, 1.94; 95% CI, 1.44–2.62), physical activity (OR, 1.68; 95% CI, 1.25–2.27), stress (OR, 1.47; 95% CI, 1.02–2.15), and unmarried status (OR, 13.60; 95% CI, 8.82–21.57), and was inversely associated with female gender (OR, 0.49; 95% CI, 0.37–0.66), overweight (OR, 0.43; 95% CI, 0.27–0.68) and obese BMI (OR, 0.36; 95% CI, 0.25–0.52), first-degree family history (OR, 0.37; 95% CI, 0.27–0.50), and hypertension (OR, 0.58; 95% CI, 0.40–0.83). Education, unemployment (n = 723), and tobacco use were not associated with diabetes type.

In the multivariable model, older age at diagnosis remained strongly associated with lower odds of type 1 diabetes (aOR, 0.0767; 95% CI, 0.0083–0.3134 for 11–20 years; aOR, 0.0083; 95% CI, 0.0009–0.0335 for 21–30 years; and aOR, 0.0034; 95% CI, 0.0004–0.0144 for 31–40 years; all *p* < 0.001), as was female gender (aOR, 0.35; 95% CI, 0.24–0.51; *p* < 0.001). Parental consanguinity (aOR, 1.98; 95% CI, 1.36–2.91; *p* < 0.001) and underweight BMI (aOR, 1.95; 95% CI, 1.04–3.70; *p* = 0.038) were associated with higher odds of type 1 diabetes. Overweight (aOR, 0.92; 95% CI, 0.52–1.62) and obese BMI (aOR, 0.88; 95% CI, 0.56–1.41), first-degree family history (aOR, 0.94; 95% CI, 0.61–1.48; *p* = 0.800), and physical activity (aOR, 1.35; 95% CI, 0.92–1.98; *p* = 0.122) were not associated with diabetes type after adjustment.

Generalized variance inflation factors ranged from 1.01 to 1.21, indicating no relevant multicollinearity. The Hosmer–Lemeshow test showed no evidence of poor calibration (χ^2^ = 2.36, df = 8, *p* = 0.968), and the calibration plot showed close agreement between predicted and observed proportions (Supplementary Figure S1), with an optimism-corrected calibration slope of 0.98. The model had an apparent AUC of 0.854 (95% CI, 0.824–0.883), with a sensitivity of 73.3% and a specificity of 85.5% at the optimal threshold, and an optimism-corrected AUC of 0.846. A model containing age at diagnosis alone had an AUC of 0.798 (95% CI, 0.765–0.831; *p* < 0.001 for the difference) (Supplementary Figure S2).

In sensitivity analyses (Supplementary Table S2), the associations of age at diagnosis, female gender, and parental consanguinity were consistent when the two youngest groups were combined into a single reference category (consanguinity aOR, 1.98; 95% CI, 1.36–2.89), when participants younger than 18 years were excluded (aOR, 2.04; 95% CI, 1.39–3.02), when the model was restricted to fixed or pre-diagnosis characteristics (aOR, 2.04; 95% CI, 1.40–3.00), when family history in any relative replaced first-degree family history (aOR, 1.99; 95% CI, 1.37–2.92), and when education was added (aOR, 1.96; 95% CI, 1.35–2.88). The main findings were also unchanged when both education and occupation were added (consanguinity aOR, 2.10; 95% CI, 1.42–3.14; first-degree family history aOR, 0.87; 95% CI, 0.55–1.40). In this model, the inverse association with female gender was stronger (aOR, 0.18; 95% CI, 0.11–0.29) because unemployment was concentrated among women, and unemployment itself was associated with type 1 diabetes (aOR, 4.22; 95% CI, 2.53–7.18). A complete-case analysis restricted to participants with known employment status (n = 722) gave similar results (consanguinity aOR, 2.52; 95% CI, 1.64–3.95). After exclusion of the 66 participants with clinically assigned type 1 diabetes who were treated with oral agents only, the association with consanguinity was attenuated but remained statistically significant (aOR, 1.62; 95% CI, 1.02–2.59; *p* = 0.042). The association with underweight BMI was not robust: it was no longer statistically significant with the combined reference category (aOR, 1.74; 95% CI, 0.93–3.28), after exclusion of participants younger than 18 years (aOR, 1.86; 95% CI, 0.98–3.55), or after adjustment for education and occupation (aOR, 1.78; 95% CI, 0.90–3.50), but was stronger after exclusion of these 66 participants (aOR, 2.34; 95% CI, 1.16–4.76). First-degree family history was not associated with diabetes type in any sensitivity analysis.

### 3.5. Comparison of Clinically Assigned Type 1 and Type 2 Diabetes

Compared with participants with type 2 diabetes, those with clinically assigned type 1 diabetes were younger at diagnosis (19.3 ± 8.6 vs. 29.7 ± 5.6 years) and at enrollment, less often female (47.5% vs. 64.7%), and more often had consanguineous parents (62.0% vs. 45.6%; first-cousin unions, 44.7% vs. 31.3%) (Table 5; all *p* < 0.001). They less often reported diabetes in a first-degree relative (56.5% vs. 78.0%), but when a relative was affected, that relative more often had type 1 diabetes (46.9% vs. 10.3%). Participants with type 1 diabetes had lower BMI (22.2 ± 5.3 vs. 25.1 ± 4.7 kg/m^2^) and blood pressure, whereas HbA1c did not differ (9.2 ± 2.4% vs. 9.1 ± 2.3%; *p* = 0.506). Treatment did not always match the clinical label: 66 participants (25.9%) with type 1 diabetes were treated with oral agents only, and 155 (25.8%) with type 2 diabetes were treated with insulin only.

## 4. Discussion

In this cross-sectional study of early-onset diabetes in Lahore and Kasur, the distribution of clinically assigned diabetes type differed markedly by age at diagnosis: type 1 diabetes accounted for most cases diagnosed at 1–20 years and type 2 diabetes for most cases diagnosed at 21–40 years. Because participants were compared across age-at-diagnosis groups at a single time point, these differences describe associations between age at onset and clinical presentation, not a transition within individuals or a change over time. The pattern is consistent with evidence that age at diagnosis is the clinical feature that best discriminates type 1 from type 2 diabetes [13] and with the increasing occurrence of type 2 diabetes among adolescents and young adults, particularly in populations with strong familial predisposition and rapid lifestyle change [8,14,36,37,38]. South Asians develop type 2 diabetes at lower levels of adiposity than White populations, which has been attributed to reduced β-cell reserve and high sensitivity to weight gain [12,16,39]. Although BMI and blood pressure were higher in later-onset groups, glycemic control was similarly poor in all groups, indicating that BMI alone does not capture metabolic severity in this population.

The most notable finding is the independent association between parental consanguinity and clinically assigned type 1 diabetes, which persisted after adjustment for age at diagnosis, gender, BMI, family history, and physical activity, and in all sensitivity analyses. Consanguinity increases homozygosity for recessive variants [20], and recessive forms of monogenic and syndromic diabetes are well documented in consanguineous Pakistani families, often presenting as insulin-requiring diabetes in childhood [21]. Because type 1 diabetes in this study was a clinical label, some cases associated with consanguinity may represent monogenic or syndromic diabetes rather than autoimmune diabetes; the attenuation of the association after exclusion of participants whose clinical label was discordant with their treatment is compatible with this interpretation. Consanguinity also increases the sharing of HLA and non-HLA susceptibility alleles within families [19]. Our data cannot distinguish these explanations, but they suggest that genetic evaluation should be considered in young patients from consanguineous families, particularly when the phenotype is atypical.

Family history was not independently associated with diabetes type. Diabetes in a first-degree relative was more common among participants with type 2 diabetes (78.0% vs. 56.5%), as expected given the strong familial aggregation of early-onset type 2 diabetes [8,9], but this difference was explained by age at diagnosis and the other covariates. Diabetes in at least one relative was reported by 84.0% of participants, reflecting the high background prevalence of diabetes in Pakistan, where 26.3% of adults have diabetes [6], as well as large multigenerational families and frequent consanguineous marriage; family history therefore has limited value for distinguishing diabetes types in this setting. A recent multicenter study from Sindh similarly found strong associations of early-onset diabetes with family history, physical inactivity, sugary beverage intake, and abnormal sleep duration [40]. Nevertheless, the type of diabetes in affected relatives differed sharply between the two groups, and the high prevalence of familial diabetes supports screening of relatives of patients with early-onset diabetes.

Underweight BMI was associated with clinically assigned type 1 diabetes, as expected given that body habitus informed the clinical classification, but this association was not robust in sensitivity analyses. Overweight and obesity were not independently associated with diabetes type after adjustment for age at diagnosis, which does not exclude a role for adiposity in early-onset type 2 diabetes; in South Asians, conventional BMI thresholds may not adequately reflect metabolic risk [17,18]. Physical inactivity was highly prevalent, and walking frequency declined with older age at diagnosis, yet physical activity was not independently associated with diabetes type; because lifestyle was assessed after diagnosis, these comparisons describe current behavior and cannot be interpreted as risk factors. Short sleep was more frequent in older groups, which is directionally consistent with meta-analytic evidence linking short sleep to type 2 diabetes [22], and tobacco use increased with older age at diagnosis, consistent with evidence linking smoking to type 2 diabetes [24,25]; neither was included in the multivariable model.

One apparently paradoxical observation was the absence of differences in fasting glucose, random glucose, and HbA1c across age-at-diagnosis groups despite marked differences in BMI, blood pressure, and diabetes type. This probably reflects the fact that all participants had established diabetes, and most were already receiving treatment; once overt hyperglycemia is established, cross-sectional glycemic markers may converge across distinct etiological pathways. The similarity in HbA1c should therefore not be taken to imply biological similarity; rather, it indicates a shared burden of suboptimal glycemic control across the entire early-onset spectrum. This is concerning because early-onset diabetes, whether type 1 or type 2, is associated with longer lifetime exposure to hyperglycemia and earlier complications [9,11,41,42].

Some misclassification of diabetes type is likely; for example, 66 participants (25.9%) with clinically assigned type 1 diabetes were treated with oral agents only. Type 1 diabetes diagnosed after 30 years of age is difficult to recognize clinically and is often treated as type 2 diabetes [43,44]. Because age and body habitus contributed to the clinical classification, misclassification is probably differential, which would inflate the associations of age at diagnosis and underweight BMI with type 1 diabetes and the discrimination of the model.

Insulin resistance is central to early-onset type 2 diabetes in South Asians, who develop diabetes at a lower BMI than White populations because of greater visceral and ectopic fat, lower muscle mass, and possibly reduced β-cell reserve [16,39]. Diabetes classified clinically as type 2 is also metabolically heterogeneous: data-driven clustering has identified insulin-deficient and insulin-resistant subgroups with different risks of complications [45], and in Asian Indians a distinct subgroup combining insulin resistance and insulin deficiency has been described [46]. Experimental work in mice has also shown that dietary polysaccharides can improve the regulation of glycolipid metabolism and alter the gut microbiome [47], pointing to potentially modifiable metabolic pathways. The higher BMI and blood pressure observed with later age at diagnosis in our cohort are consistent with an increasing contribution of insulin resistance, but because insulin, C-peptide, and lipid measurements were not available, we could not assess insulin resistance or β-cell function directly.

The multivariable model discriminated well, but most of its discrimination came from age at diagnosis, which, together with body habitus, informed the clinical assignment of diabetes type; the AUC therefore partly reflects the classification process itself. The model should be interpreted as describing correlates of the clinical label, not as a tool for classifying diabetes type, and external validation against autoantibody- or C-peptide-confirmed diagnoses would be required before any clinical use.

### Strengths and Limitations

The strengths of this study include its multicenter design, the comparison of four age-at-diagnosis groups from childhood to young adulthood, and the combination of familial, anthropometric, behavioral, and clinical data. It also has important limitations. First, diabetes type was assigned clinically, on the basis of insulin requirement, diabetic ketoacidosis at presentation, and body habitus, without systematic autoantibody, C-peptide, or genetic testing. Clinical features discriminate imperfectly between type 1 and type 2 diabetes [13]. Secondly, lifestyle characteristics, BMI, and blood pressure were assessed at enrollment, on average about 6 years after diagnosis, and may have been modified by the diagnosis, its treatment (for example, insulin-associated weight gain), or lifestyle advice; associations involving these variables cannot be interpreted as risk factors for either type of diabetes. Thirdly, the cross-sectional design precludes causal inference, and the hospital-based sample may not represent the general population. Residual confounding by socioeconomic factors cannot be excluded, although adjustment for education and occupation did not change the main findings. Finally, monogenic diabetes was not systematically excluded, and BMI may not adequately reflect metabolic risk in South Asians.

## 5. Conclusions

In this hospital-based cohort from Lahore and Kasur, the distribution of clinically assigned diabetes type differed markedly by age at diagnosis: type 1 diabetes predominated among patients diagnosed before 20 years of age and type 2 diabetes among those diagnosed at 21–40 years. Later age at diagnosis was accompanied by higher BMI and blood pressure and by a lower prevalence of parental consanguinity, whereas glycemic control was uniformly poor across all groups. Younger age at diagnosis, male gender, parental consanguinity, and underweight BMI were independently associated with a clinical diagnosis of type 1 diabetes, whereas family history was not. Wider access to autoantibody and C-peptide testing, together with genetic evaluation of young patients from consanguineous families, is needed to establish the etiological composition of early-onset diabetes in Pakistan and to guide treatment and screening.

## Figures and Tables

**Table 1:** Demographic and family characteristics of patients with early-onset diabetes by age at diagnosis.

Variable	Overall	1–10 Years	11–20 Years	21–30 Years	31–40 Years	*p*-Value
	n = 856	n = 42	n = 140	n = 356	n = 318	
District						0.672
Kasur	137 (16.0%)	8 (19.0%)	18 (12.9%)	57 (16.0%)	54 (17.0%)	
Lahore	719 (84.0%)	34 (81.0%)	122 (87.1%)	299 (84.0%)	264 (83.0%)	
Age at diagnosis (years)	26.6 ± 8.2	6.8 ± 2.7	16.0 ± 2.6	26.2 ± 2.8	34.3 ± 2.3	<0.001
Age at enrollment (years)	32.5 ± 7.4	20.5 ± 6.7	23.2 ± 6.2	32.6 ± 5.1	37.9 ± 3.2	<0.001
Diabetes type						<0.001
Type 1	255 (29.8%)	41 (97.6%)	103 (73.6%)	78 (21.9%)	33 (10.4%)	
Type 2	601 (70.2%)	1 (2.4%)	37 (26.4%)	278 (78.1%)	285 (89.6%)	
Parental relationship						0.019
1st Cousin	302 (35.3%)	18 (42.9%)	59 (42.1%)	139 (39.0%)	86 (27.0%)	
2nd Cousin	35 (4.1%)	1 (2.4%)	3 (2.1%)	14 (3.9%)	17 (5.3%)	
3rd Cousin	22 (2.6%)	0 (0.0%)	4 (2.9%)	5 (1.4%)	13 (4.1%)	
Blood relation only	73 (8.5%)	6 (14.3%)	11 (7.9%)	26 (7.3%)	30 (9.4%)	
No family relation	424 (49.5%)	17 (40.5%)	63 (45.0%)	172 (48.3%)	172 (54.1%)	
Family history of diabetes (any relative)	719 (84.0%)	28 (66.7%)	104 (74.3%)	309 (86.8%)	278 (87.4%)	<0.001
Family history of diabetes (first-degree relative)	613 (71.6%)	13 (31.0%)	69 (49.3%)	276 (77.5%)	255 (80.2%)	<0.001
Diabetes type in affected relative (s)						0.068
Type 1	146 (20.3%)	11 (39.3%)	24 (23.1%)	67 (21.7%)	44 (15.8%)	
Type 2	564 (78.4%)	17 (60.7%)	78 (75.0%)	238 (77.0%)	231 (83.1%)	
Not recorded	9 (1.3%)	0 (0.0%)	2 (1.9%)	4 (1.3%)	3 (1.1%)	
Gender						0.158
Female	510 (59.6%)	30 (71.4%)	76 (54.3%)	220 (61.8%)	184 (57.9%)	
Male	346 (40.4%)	12 (28.6%)	64 (45.7%)	136 (38.2%)	134 (42.1%)	
Highest education						0.010
College/University completed	103 (12.0%)	1 (2.4%)	20 (14.3%)	50 (14.0%)	32 (10.1%)	
High school completed	139 (16.2%)	7 (16.7%)	21 (15.0%)	66 (18.5%)	45 (14.2%)	
No formal schooling	169 (19.7%)	7 (16.7%)	18 (12.9%)	74 (20.8%)	70 (22.0%)	
Post graduate degree	15 (1.8%)	1 (2.4%)	5 (3.6%)	4 (1.1%)	5 (1.6%)	
Primary school completed	193 (22.5%)	17 (40.5%)	32 (22.9%)	61 (17.1%)	83 (26.1%)	
Refused	1 (0.1%)	0 (0.0%)	0 (0.0%)	1 (0.3%)	0 (0.0%)	
Secondary school completed	236 (27.6%)	9 (21.4%)	44 (31.4%)	100 (28.1%)	83 (26.1%)	
Ethnicity						0.605
Pathan	5 (0.6%)	0 (0.0%)	1 (0.7%)	1 (0.3%)	3 (0.9%)	
Punjabi	844 (98.6%)	42 (100.0%)	137 (97.9%)	351 (98.6%)	314 (98.7%)	
Refused	7 (0.8%)	0 (0.0%)	2 (1.4%)	4 (1.1%)	1 (0.3%)	
Marital status						<0.001
Divorced	1 (0.1%)	0 (0.0%)	1 (0.7%)	0 (0.0%)	0 (0.0%)	
Married	722 (84.3%)	13 (31.0%)	70 (50.0%)	328 (92.1%)	311 (97.8%)	
Unmarried	133 (15.5%)	29 (69.0%)	69 (49.3%)	28 (7.9%)	7 (2.2%)	
Employment						<0.001
Government employee	32 (3.7%)	0 (0.0%)	0 (0.0%)	12 (3.4%)	20 (6.3%)	
Non-government employee	121 (14.1%)	3 (7.1%)	31 (22.1%)	45 (12.6%)	42 (13.2%)	
Others	71 (8.3%)	2 (4.8%)	2 (1.4%)	35 (9.8%)	32 (10.1%)	
No answer	56 (6.5%)	4 (9.5%)	2 (1.4%)	26 (7.3%)	24 (7.5%)	
Self-employed	141 (16.5%)	4 (9.5%)	25 (17.9%)	59 (16.6%)	53 (16.7%)	
Student	77 (9.0%)	15 (35.7%)	40 (28.6%)	17 (4.8%)	5 (1.6%)	
Unemployed	358 (41.8%)	14 (33.3%)	40 (28.6%)	162 (45.5%)	142 (44.7%)	

Values are n (%) or mean ± SD. *p*-values were calculated using the chi-square test, or Fisher’s exact test when more than 20% of expected cell counts were below 5 (Monte Carlo simulation for tables larger than 2 × k), for categorical variables and Welch’s ANOVA for continuous variables. First-degree relative: parent or sibling. The type of diabetes in affected relatives is shown for the 719 participants with an affected relative.

**Table 2:** Lifestyle characteristics of patients with early-onset diabetes by age at diagnosis.

Variable	Overall	1–10 Years	11–20 Years	21–30 Years	31–40 Years	*p*-Value
	n = 856	n= 42	n = 140	n = 356	n = 318	
Tobacco smoking	51 (6.0%)	0 (0.0%)	3 (2.1%)	19 (5.3%)	29 (9.1%)	0.007
Smokeless tobacco	41 (4.8%)	1 (2.4%)	9 (6.4%)	14 (3.9%)	17 (5.3%)	0.542
Ever tobacco use	71 (8.3%)	0 (0.0%)	9 (6.4%)	27 (7.6%)	35 (11.0%)	0.051
Sugary beverages (servings/week)						0.402
0	442 (51.6%)	17 (40.5%)	70 (50.0%)	181 (50.8%)	174 (54.7%)	
1	222 (25.9%)	12 (28.6%)	32 (22.9%)	94 (26.4%)	84 (26.4%)	
2	111 (13.0%)	6 (14.3%)	22 (15.7%)	47 (13.2%)	36 (11.3%)	
3	28 (3.3%)	3 (7.1%)	4 (2.9%)	10 (2.8%)	11 (3.5%)	
4	7 (0.8%)	1 (2.4%)	1 (0.7%)	2 (0.6%)	3 (0.9%)	
5	1 (0.1%)	0 (0.0%)	0 (0.0%)	0 (0.0%)	1 (0.3%)	
6	2 (0.2%)	0 (0.0%)	0 (0.0%)	1 (0.3%)	1 (0.3%)	
7	43 (5.0%)	3 (7.1%)	11 (7.9%)	21 (5.9%)	8 (2.5%)	
Fruit consumption (days/week)						0.007
0	52 (6.1%)	6 (14.3%)	13 (9.3%)	19 (5.3%)	14 (4.4%)	
1	60 (7.0%)	2 (4.8%)	9 (6.4%)	27 (7.6%)	22 (6.9%)	
2	220 (25.7%)	8 (19.0%)	29 (20.7%)	98 (27.5%)	85 (26.7%)	
3	261 (30.5%)	8 (19.0%)	38 (27.1%)	99 (27.8%)	116 (36.5%)	
4	85 (9.9%)	6 (14.3%)	9 (6.4%)	38 (10.7%)	32 (10.1%)	
5	23 (2.7%)	1 (2.4%)	4 (2.9%)	11 (3.1%)	7 (2.2%)	
6	10 (1.2%)	0 (0.0%)	1 (0.7%)	3 (0.8%)	6 (1.9%)	
7	145 (16.9%)	11 (26.2%)	37 (26.4%)	61 (17.1%)	36 (11.3%)	
Vegetable consumption (days/week)						<0.001
0	8 (0.9%)	0 (0.0%)	2 (1.4%)	4 (1.1%)	2 (0.6%)	
1	6 (0.7%)	0 (0.0%)	3 (2.1%)	2 (0.6%)	1 (0.3%)	
2	62 (7.2%)	1 (2.4%)	6 (4.3%)	25 (7.0%)	30 (9.4%)	
3	248 (29.0%)	11 (26.2%)	29 (20.7%)	103 (28.9%)	105 (33.0%)	
4	195 (22.8%)	6 (14.3%)	33 (23.6%)	88 (24.7%)	68 (21.4%)	
5	92 (10.7%)	2 (4.8%)	12 (8.6%)	32 (9.0%)	46 (14.5%)	
6	42 (4.9%)	1 (2.4%)	3 (2.1%)	20 (5.6%)	18 (5.7%)	
7	203 (23.7%)	21 (50.0%)	52 (37.1%)	82 (23.0%)	48 (15.1%)	
Junk food consumption						0.016
Daily	13 (1.5%)	1 (2.4%)	3 (2.1%)	4 (1.1%)	5 (1.6%)	
Never	145 (16.9%)	9 (21.4%)	16 (11.4%)	58 (16.3%)	62 (19.5%)	
Once in a month	300 (35.0%)	13 (31.0%)	49 (35.0%)	121 (34.0%)	117 (36.8%)	
Once in a week	86 (10.0%)	0 (0.0%)	21 (15.0%)	45 (12.6%)	20 (6.3%)	
Rarely	283 (33.1%)	19 (45.2%)	44 (31.4%)	114 (32.0%)	106 (33.3%)	
Twice a week	29 (3.4%)	0 (0.0%)	7 (5.0%)	14 (3.9%)	8 (2.5%)	
Walking (days/week)						0.003
0	552 (64.5%)	22 (52.4%)	77 (55.0%)	233 (65.4%)	220 (69.2%)	
1	7 (0.8%)	0 (0.0%)	1 (0.7%)	2 (0.6%)	4 (1.3%)	
2	23 (2.7%)	3 (7.1%)	5 (3.6%)	9 (2.5%)	6 (1.9%)	
3	30 (3.5%)	0 (0.0%)	7 (5.0%)	19 (5.3%)	4 (1.3%)	
4	15 (1.8%)	1 (2.4%)	0 (0.0%)	8 (2.2%)	6 (1.9%)	
5	19 (2.2%)	1 (2.4%)	1 (0.7%)	8 (2.2%)	9 (2.8%)	
6	4 (0.5%)	0 (0.0%)	2 (1.4%)	2 (0.6%)	0 (0.0%)	
7	206 (24.1%)	15 (35.7%)	47 (33.6%)	75 (21.1%)	69 (21.7%)	
Sports activity (days/week)						0.551
0	823 (96.1%)	39 (92.9%)	137 (97.9%)	339 (95.2%)	308 (96.9%)	
1	3 (0.4%)	0 (0.0%)	0 (0.0%)	2 (0.6%)	1 (0.3%)	
2	2 (0.2%)	0 (0.0%)	0 (0.0%)	1 (0.3%)	1 (0.3%)	
3	3 (0.4%)	0 (0.0%)	1 (0.7%)	2 (0.6%)	0 (0.0%)	
4	1 (0.1%)	1 (2.4%)	0 (0.0%)	0 (0.0%)	0 (0.0%)	
5	3 (0.4%)	0 (0.0%)	0 (0.0%)	1 (0.3%)	2 (0.6%)	
6	4 (0.5%)	1 (2.4%)	0 (0.0%)	2 (0.6%)	1 (0.3%)	
7	17 (2.0%)	1 (2.4%)	2 (1.4%)	9 (2.5%)	5 (1.6%)	
Sleep duration (per day)						0.004
6–8 h	776 (90.7%)	39 (92.9%)	129 (92.1%)	324 (91.0%)	284 (89.3%)	
Greater than 8 h	24 (2.8%)	3 (7.1%)	4 (2.9%)	14 (3.9%)	3 (0.9%)	
Less than 6 h	56 (6.5%)	0 (0.0%)	7 (5.0%)	18 (5.1%)	31 (9.7%)	
Bedtime						0.183
Early (8:00–10:00 pm)	761 (88.9%)	39 (92.9%)	131 (93.6%)	311 (87.4%)	280 (88.1%)	
Late (after 10:30 pm)	95 (11.1%)	3 (7.1%)	9 (6.4%)	45 (12.6%)	38 (11.9%)	

Values are n (%). *p*-values were calculated using the chi-square test, or Fisher’s exact test when more than 20% of expected cell counts were below 5 (Monte Carlo simulation for tables larger than 2 × k).

**Table 3:** Clinical and anthropometric characteristics of patients with early-onset diabetes by age at diagnosis.

Variable	Overall	1–10 Years	11–20 Years	21–30 Years	31–40 Years	*p*-Value
	n = 856	n = 42	n = 140	n = 356	n = 318	
Experience of stress	666 (77.8%)	36 (85.7%)	104 (74.3%)	273 (76.7%)	253 (79.6%)	0.341
Antidiabetic treatment						<0.001
Both Insulin and Tablets	192 (22.4%)	1 (2.4%)	19 (13.6%)	99 (27.8%)	73 (23.0%)	
Insulin only	322 (37.6%)	40 (95.2%)	104 (74.3%)	122 (34.3%)	56 (17.6%)	
Tablets only	342 (40.0%)	1 (2.4%)	17 (12.1%)	135 (37.9%)	189 (59.4%)	
Antihypertensive drugs						0.005
Yes	81 (9.5%)	2 (4.8%)	3 (2.1%)	38 (10.7%)	38 (11.9%)	
No	775 (90.5%)	40 (95.2%)	137 (97.9%)	318 (89.3%)	280 (88.1%)	
Cardiovascular preventive drugs						0.507
Aspirin	13 (1.5%)	0 (0.0%)	1 (0.7%)	3 (0.8%)	9 (2.8%)	
Nil	835 (97.5%)	42 (100.0%)	138 (98.6%)	349 (98.0%)	306 (96.2%)	
Statins	8 (0.9%)	0 (0.0%)	1 (0.7%)	4 (1.1%)	3 (0.9%)	
Systolic blood pressure (mmHg)	122.0 ± 15.5	111.5 ± 15.4	113.3 ± 12.5	123.3 ± 16.0	125.9 ± 14.0	<0.001
Diastolic blood pressure (mmHg)	78.1 ± 9.4	71.4 ± 9.7	73.9 ± 9.2	78.4 ± 9.6	80.4 ± 8.3	<0.001
Hypertension	224 (26.2%)	6 (14.3%)	16 (11.4%)	101 (28.4%)	101 (31.8%)	<0.001
Height (cm)	162.0 ± 9.8	154.6 ± 13.4	161.0 ± 11.6	163.0 ± 8.4	162.3 ± 9.5	<0.001
Weight (kg)	63.6 ± 13.7	46.9 ± 10.2	55.8 ± 14.5	64.8 ± 12.3	67.9 ± 12.3	<0.001
BMI (kg/m^2^)	24.3 ± 5.0	19.5 ± 3.0	21.4 ± 4.8	24.5 ± 4.7	25.9 ± 4.8	<0.001
BMI category						<0.001
Underweight	80 (9.3%)	5 (11.9%)	32 (22.9%)	30 (8.4%)	13 (4.1%)	
Normal	295 (34.5%)	34 (81.0%)	69 (49.3%)	114 (32.0%)	78 (24.5%)	
Overweight	144 (16.8%)	1 (2.4%)	14 (10.0%)	79 (22.2%)	50 (15.7%)	
Obese	337 (39.4%)	2 (4.8%)	25 (17.9%)	133 (37.4%)	177 (55.7%)	
Fasting Blood Glucose (mg/dL)	167.2 ± 48.3	165.5 ± 31.1	166.4 ± 53.1	170.0 ± 53.7	164.5 ± 41.1	0.520
Random blood glucose (mg/dL)	287.7 ± 106.4	313.1 ± 113.7	284.8 ± 116.6	289.0 ± 104.7	284.1 ± 102.7	0.460
HbA1c (%)	9.1 ± 2.3	9.5 ± 2.2	9.3 ± 2.4	9.1 ± 2.5	9.0 ± 2.2	0.356

Values are n (%) or mean ± SD. *p*-values were calculated using the chi-square or Fisher’s exact test for categorical variables and Welch’s ANOVA for continuous variables; Games–Howell pairwise comparisons are given in Supplementary Table S1. Hypertension: blood pressure ≥ 140/90 mmHg or antihypertensive treatment. BMI category: WHO cut-offs for Asian populations in participants aged ≥18 years and WHO BMI-for-age z-scores in those aged <18 years. Glycemic indicators were measured at enrollment and reflect current glycemic control, not etiologic subtype or baseline β-cell phenotype.

**Table 4:** Univariable and multivariable Firth logistic regression of factors associated with clinically assigned type 1 diabetes (reference: type 2 diabetes).

Variable	n	Events	Univariable Analysis		Multivariable Analysis	
			OR (95% CI)	*p*-Value	aOR (95% CI)	*p*-Value
Age at diagnosis	856	255				
1–10 years (Ref)			—		—	
11–20 years			0.0998 (0.0110–0.3949)	<0.001	0.0767 (0.0083–0.3134)	<0.001
21–30 years			0.0102 (0.0011–0.0389)	<0.001	0.0083 (0.0009–0.0335)	<0.001
31–40 years			0.0042 (0.0005–0.0167)	<0.001	0.0034 (0.0004–0.0144)	<0.001
Gender	856	255				
Male (Ref)			—		—	
Female			0.49 (0.37–0.66)	<0.001	0.35 (0.24–0.51)	<0.001
BMI category	856	255				
Normal (Ref)			—		—	
Underweight			2.11 (1.28–3.49)	0.003	1.95 (1.04–3.70)	0.038
Overweight			0.43 (0.27–0.68)	<0.001	0.92 (0.52–1.62)	0.784
Obese			0.36 (0.25–0.52)	<0.001	0.88 (0.56–1.41)	0.604
First-degree family history	856	255				
No (Ref)			—		—	
Yes			0.37 (0.27–0.50)	<0.001	0.94 (0.61–1.48)	0.800
Parental consanguinity	856	255				
No (Ref)			—		—	
Yes			1.94 (1.44–2.62)	<0.001	1.98 (1.36–2.91)	<0.001
Education	855	255				
Low (Ref)			—		—	
Medium			1.17 (0.85–1.61)	0.328	—	—
High			1.19 (0.75–1.85)	0.461	—	—
Physical activity	856	255				
Inactive (Ref)			—		—	
Active			1.68 (1.25–2.27)	<0.001	1.35 (0.92–1.98)	0.122
Occupation	723	178				
Employed (Ref)			—		—	
Unemployed			1.23 (0.87–1.72)	0.237	—	—
Marital status	856	255				
Married (Ref)			—		—	
Unmarried			13.60 (8.82–21.57)	<0.001	—	—
Stress	856	255				
No (Ref)			—		—	
Yes			1.47 (1.02–2.15)	0.036	—	—
Tobacco use	856	255				
No (Ref)			—		—	
Yes			0.65 (0.32–1.24)	0.197	—	—
Hypertension	856	255				
No (Ref)			—		—	
Yes			0.58 (0.40–0.83)	0.002	—	—

OR, odds ratio; aOR, adjusted odds ratio; CI, confidence interval; Ref, reference category; —, not included in the multivariable model. ORs, profile penalized-likelihood 95% CIs, and *p*-values were estimated by Firth’s penalized logistic regression. The multivariable model (856 participants, 255 with type 1 diabetes, 10 parameters) was adjusted for age at diagnosis, gender, BMI category, first-degree family history, parental consanguinity, and physical activity. Only 1 of 42 participants diagnosed at 1–10 years had type 2 diabetes; the ORs for age at diagnosis are therefore very small and are reported to four decimal places. BMI category: WHO cut-offs for Asian populations (≥18 years) or WHO BMI-for-age z-scores (<18 years). Physical activity: walking or sports on at least one day per week. Hypertension: blood pressure ≥ 140/90 mmHg or antihypertensive treatment.

**Table 5:** Characteristics of participants by clinically assigned diabetes type.

Variable	Overall	Type 1 Diabetes	Type 2 Diabetes	*p*-Value
	n = 856	n = 255	n = 601	
Age at diagnosis (years)	26.6 ± 8.2	19.3 ± 8.6	29.7 ± 5.6	<0.001
Age at enrollment (years)	32.5 ± 7.4	25.7 ± 8.0	35.3 ± 4.9	<0.001
Female gender	510 (59.6%)	121 (47.5%)	389 (64.7%)	<0.001
Parental consanguinity	432 (50.5%)	158 (62.0%)	274 (45.6%)	<0.001
Parental relationship				<0.001
1st Cousin	302 (35.3%)	114 (44.7%)	188 (31.3%)	
2nd Cousin	35 (4.1%)	7 (2.7%)	28 (4.7%)	
3rd Cousin	22 (2.6%)	9 (3.5%)	13 (2.2%)	
Blood relation only	73 (8.5%)	28 (11.0%)	45 (7.5%)	
No family relation	424 (49.5%)	97 (38.0%)	327 (54.4%)	
Family history (any relative)	719 (84.0%)	196 (76.9%)	523 (87.0%)	<0.001
Family history (first-degree relative)	613 (71.6%)	144 (56.5%)	469 (78.0%)	<0.001
Diabetes type in affected relative (s)				<0.001
Type 1	146 (20.3%)	92 (46.9%)	54 (10.3%)	
Type 2	564 (78.4%)	101 (51.5%)	463 (88.5%)	
Not recorded	9 (1.3%)	3 (1.5%)	6 (1.1%)	
BMI (kg/m^2^)	24.3 ± 5.0	22.2 ± 5.3	25.1 ± 4.7	<0.001
BMI category				<0.001
Underweight	80 (9.3%)	46 (18.0%)	34 (5.7%)	
Normal	295 (34.5%)	115 (45.1%)	180 (30.0%)	
Overweight	144 (16.8%)	31 (12.2%)	113 (18.8%)	
Obese	337 (39.4%)	63 (24.7%)	274 (45.6%)	
Physically active	309 (36.1%)	114 (44.7%)	195 (32.4%)	<0.001
Antidiabetic treatment				<0.001
Insulin only	322 (37.6%)	167 (65.5%)	155 (25.8%)	
Tablets only	342 (40.0%)	66 (25.9%)	276 (45.9%)	
Insulin and tablets	192 (22.4%)	22 (8.6%)	170 (28.3%)	
Systolic BP (mmHg)	122.0 ± 15.5	116.1 ± 13.4	124.5 ± 15.6	<0.001
Diastolic BP (mmHg)	78.1 ± 9.4	74.9 ± 9.2	79.4 ± 9.2	<0.001
Hypertension	224 (26.2%)	49 (19.2%)	175 (29.1%)	0.003
Fasting blood glucose (mg/dL)	167.2 ± 48.3	163.2 ± 44.5	168.9 ± 49.8	0.101
Random blood glucose (mg/dL)	287.7 ± 106.4	292.0 ± 111.4	285.9 ± 104.3	0.457
HbA1c (%)	9.1 ± 2.3	9.2 ± 2.4	9.1 ± 2.3	0.506

Values are n (%) or mean ± SD. *p*-values were calculated using Welch’s *t*-test for continuous variables and the chi-square test for categorical variables. The type of diabetes in affected relatives is shown for participants with an affected relative (n = 719). BP, blood pressure.

## Data Availability

The data presented in this study are available on request from the corresponding authors. The data are not publicly available because they contain personal information about the participants.

## Supplementary Materials

The following supporting information can be downloaded at: https://www.mdpi.com/article/10.3390/jcm15218390/s1, Table S1: Games–Howell pairwise comparisons of continuous variables across age-at-diagnosis groups; Table S2: Sensitivity analyses of factors associated with clinically assigned type 1 diabetes; Figure S1: Calibration plot of the multivariable model; Figure S2: Receiver operating characteristic curves of the multivariable model and of age at diagnosis alone.
