# Supplementary material for: Early-Onset Diabetes in Lahore and Kasur Districts of Punjab: A Hospital-Based Study
Source: J Clin Med. 2026 Oct 28;15(21):8390. doi: 10.3390/jcm15218390 (PMC13763600; doi:10.3390/jcm15218390)
Supplement: Supplementary file 1 [file jcm-15-08390-s001.zip › jcm-4606167-supplementary.pdf]

Supplementary Materials

Early-Onset Diabetes in Lahore and Kasur Districts of Punjab: A Hospital-Based Study

Table S1. Games–Howell pairwise comparisons of continuous variables across age-at-diagnosis groups.

Variable	Comparison	Mean difference	p-value
Age at enrollment (years)	11–20 vs 1–10 years	2.78	0.091
	21–30 vs 1–10 years	12.17	<0.001
	31–40 vs 1–10 years	17.49	<0.001
	21–30 vs 11–20 years	9.40	<0.001
	31–40 vs 11–20 years	14.71	<0.001
	31–40 vs 21–30 years	5.32	<0.001
Systolic blood pressure (mmHg)	11–20 vs 1–10 years	1.77	0.904
	21–30 vs 1–10 years	11.76	<0.001
	31–40 vs 1–10 years	14.36	<0.001
	21–30 vs 11–20 years	9.99	<0.001
	31–40 vs 11–20 years	12.59	<0.001
	31–40 vs 21–30 years	2.60	0.112
Diastolic blood pressure (mmHg)	11–20 vs 1–10 years	2.56	0.428
	21–30 vs 1–10 years	7.03	<0.001
	31–40 vs 1–10 years	8.98	<0.001
	21–30 vs 11–20 years	4.47	<0.001
	31–40 vs 11–20 years	6.42	<0.001
	31–40 vs 21–30 years	1.95	0.025
Height (cm)	11–20 vs 1–10 years	6.37	0.035
	21–30 vs 1–10 years	8.39	0.001
	31–40 vs 1–10 years	7.71	0.004
	21–30 vs 11–20 years	2.02	0.240
	31–40 vs 11–20 years	1.33	0.629
	31–40 vs 21–30 years	-0.69	0.754
Weight (kg)	11–20 vs 1–10 years	8.93	<0.001
	21–30 vs 1–10 years	17.94	<0.001
	31–40 vs 1–10 years	21.02	<0.001
	21–30 vs 11–20 years	9.01	<0.001
	31–40 vs 11–20 years	12.08	<0.001
	31–40 vs 21–30 years	3.07	0.007
BMI (kg/m^2)	11–20 vs 1–10 years	1.99	0.009
	21–30 vs 1–10 years	5.02	<0.001
	31–40 vs 1–10 years	6.43	<0.001
	21–30 vs 11–20 years	3.02	<0.001

Variable	Comparison	Mean difference	p-value
	31–40 vs 11–20 years	4.44	<0.001
	31–40 vs 21–30 years	1.41	<0.001

Mean difference = mean of the first group minus mean of the second group. p-values are adjusted for multiple comparisons by the Games–Howell procedure. All variables shown differed significantly across the four groups (Welch's ANOVA, $p < 0.001$). Fasting blood glucose, random blood glucose, and HbA1c did not differ across groups (Welch's ANOVA, $p = 0.520$, 0.460, and 0.356) and were not compared pairwise.

Table S2. Sensitivity analyses of factors associated with clinically assigned type 1 diabetes (Firth penalized logistic regression).

Model / variable	aOR (95% CI)	p-value
**Main model (n = 856; 255 with type 1 diabetes)**		
Age at diagnosis 11–20 vs 1–10 years	0.0767 (0.0083–0.3134)	<0.001
Age at diagnosis 21–30 vs 1–10 years	0.0083 (0.0009–0.0335)	<0.001
Age at diagnosis 31–40 vs 1–10 years	0.0034 (0.0004–0.0144)	<0.001
Female vs male gender	0.35 (0.24–0.51)	<0.001
Underweight vs normal BMI	1.95 (1.04–3.70)	0.038
Overweight vs normal BMI	0.92 (0.52–1.62)	0.784
Obese vs normal BMI	0.88 (0.56–1.41)	0.604
First-degree family history	0.94 (0.61–1.48)	0.800
Parental consanguinity	1.98 (1.36–2.91)	<0.001
Physically active	1.35 (0.92–1.98)	0.122
**(i) Combined ≤20-year reference category (n = 856; 255 events)**		
Age at diagnosis 21–30 vs ≤20 years	0.079 (0.048–0.126)	<0.001
Age at diagnosis 31–40 vs ≤20 years	0.033 (0.018–0.058)	<0.001
Female vs male gender	0.37 (0.25–0.54)	<0.001
Underweight vs normal BMI	1.74 (0.93–3.28)	0.086
Overweight vs normal BMI	0.84 (0.47–1.46)	0.536
Obese vs normal BMI	0.82 (0.52–1.30)	0.399
First-degree family history	0.87 (0.57–1.35)	0.538
Parental consanguinity	1.98 (1.36–2.89)	<0.001
Physically active	1.34 (0.92–1.95)	0.130
**(ii) Excluding participants younger than 18 years (n = 806; 208 events)**		
Age at diagnosis 11–20 vs 1–10 years	0.1065 (0.0114–0.4553)	0.001
Age at diagnosis 21–30 vs 1–10 years	0.0135 (0.0014–0.0564)	<0.001
Age at diagnosis 31–40 vs 1–10 years	0.0056 (0.0006–0.0245)	<0.001
Female vs male gender	0.36 (0.24–0.53)	<0.001
Underweight vs normal BMI	1.86 (0.98–3.55)	0.059
Overweight vs normal BMI	0.83 (0.46–1.48)	0.536
Obese vs normal BMI	0.86 (0.54–1.38)	0.531
First-degree family history	0.95 (0.61–1.50)	0.818
Parental consanguinity	2.04 (1.39–3.02)	<0.001
Physically active	1.28 (0.86–1.89)	0.222
**(iii) Excluding type 1 diabetes treated with oral agents only (n = 790; 189 events)**		
Age at diagnosis 11–20 vs 1–10 years	0.0687 (0.0074–0.2867)	<0.001
Age at diagnosis 21–30 vs 1–10 years	0.0070 (0.0008–0.0294)	<0.001
Age at diagnosis 31–40 vs 1–10 years	0.0010 (0.000098–0.0049)	<0.001
Female vs male gender	0.32 (0.20–0.51)	<0.001

Model / variable	aOR (95% CI)	p-value
Underweight vs normal BMI	2.34 (1.16–4.76)	0.018
Overweight vs normal BMI	0.88 (0.45–1.72)	0.721
Obese vs normal BMI	0.62 (0.34–1.12)	0.115
First-degree family history	0.81 (0.48–1.37)	0.421
Parental consanguinity	1.62 (1.02–2.59)	0.042
Physically active	1.72 (1.08–2.74)	0.023
**(iv) Fixed or pre-diagnosis characteristics only (n = 856; 255 events)**		
Age at diagnosis 11–20 vs 1–10 years	0.0815 (0.0089–0.3300)	<0.001
Age at diagnosis 21–30 vs 1–10 years	0.0079 (0.0009–0.0314)	<0.001
Age at diagnosis 31–40 vs 1–10 years	0.0031 (0.0003–0.0130)	<0.001
Female vs male gender	0.34 (0.23–0.50)	<0.001
First-degree family history	0.85 (0.56–1.30)	0.438
Parental consanguinity	2.04 (1.40–3.00)	<0.001
**(v) Family history in any relative instead of first-degree (n = 856; 255 events)**		
Age at diagnosis 11–20 vs 1–10 years	0.0755 (0.0082–0.3078)	<0.001
Age at diagnosis 21–30 vs 1–10 years	0.0080 (0.0009–0.0322)	<0.001
Age at diagnosis 31–40 vs 1–10 years	0.0033 (0.0004–0.0138)	<0.001
Female vs male gender	0.34 (0.23–0.50)	<0.001
Underweight vs normal BMI	1.98 (1.05–3.74)	0.035
Overweight vs normal BMI	0.90 (0.51–1.58)	0.725
Obese vs normal BMI	0.86 (0.55–1.38)	0.539
Family history in any relative	1.08 (0.64–1.84)	0.781
Parental consanguinity	1.99 (1.37–2.92)	<0.001
Physically active	1.36 (0.92–1.99)	0.118
**(vi) Adding education (n = 855; 255 events)**		
Age at diagnosis 11–20 vs 1–10 years	0.0747 (0.0081–0.3059)	<0.001
Age at diagnosis 21–30 vs 1–10 years	0.0082 (0.0009–0.0333)	<0.001
Age at diagnosis 31–40 vs 1–10 years	0.0035 (0.0004–0.0145)	<0.001
Female vs male gender	0.35 (0.24–0.51)	<0.001
Underweight vs normal BMI	1.93 (1.03–3.66)	0.042
Overweight vs normal BMI	0.94 (0.53–1.64)	0.822
Obese vs normal BMI	0.88 (0.55–1.40)	0.577
First-degree family history	0.92 (0.60–1.44)	0.715
Parental consanguinity	1.96 (1.35–2.88)	<0.001
Physically active	1.35 (0.92–1.98)	0.122
Education: medium vs low	1.18 (0.78–1.79)	0.420
Education: high vs low	1.17 (0.66–2.05)	0.585

aOR, adjusted odds ratio; CI, confidence interval. All models were fitted with Firth's penalized logistic regression, with profile penalized-likelihood 95% CIs and p-values. Unless stated otherwise, models were

adjusted for age at diagnosis, gender, BMI category, first-degree family history, parental consanguinity, and physical activity. Model (iii) excluded participants with clinically assigned type 1 diabetes treated with oral agents only (n = 66). Odds ratios for age at diagnosis with 1–10 years as reference are reported to four decimal places (two significant figures for values below 0.0001) because only 1 of 42 participants in the reference group had type 2 diabetes.

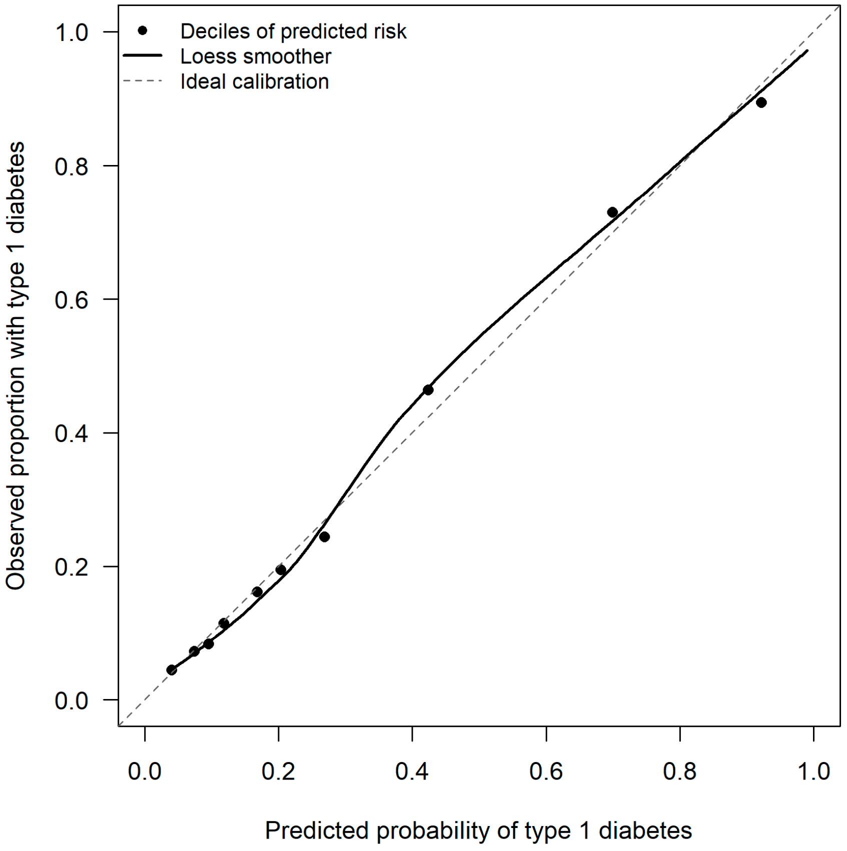

Figure S1. Calibration plot of the multivariable Firth logistic regression model for clinically assigned type 1 diabetes. Points show the observed proportion with type 1 diabetes against the mean predicted probability in deciles of predicted risk; the solid line is a loess smoother, and the dashed line indicates ideal calibration. Hosmer–Lemeshow $\chi^2 = 2.36$, df = 8, p = 0.968; apparent calibration slope 1.03 and optimism-corrected calibration slope 0.98 (1000 bootstrap replicates).

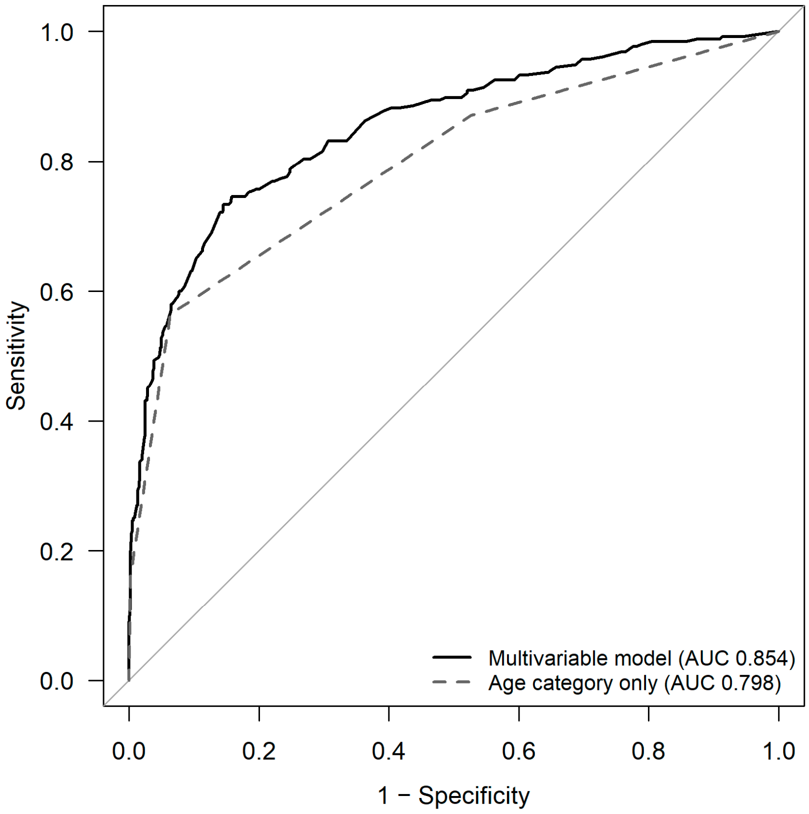

Figure S2. Receiver operating characteristic curves of the multivariable model (solid line; AUC 0.854, 95% CI 0.824–0.883; optimism-corrected AUC 0.846) and of a model containing age at diagnosis alone (dashed line; AUC 0.798, 95% CI 0.765–0.831). DeLong test for the difference, $p < 0.001$.
